# Quick assessment of influenza a virus infectivity with a long-range reverse-transcription quantitative polymerase chain reaction assay

**DOI:** 10.1186/s12879-020-05317-8

**Published:** 2020-08-06

**Authors:** Yuki Nakaya, Takashi Fukuda, Hiroki Ashiba, Masato Yasuura, Makoto Fujimaki

**Affiliations:** Sensing System Research Center, National Institute of Advanced Industrial Science and Technology (AIST), Central 5, 1-1-1 Higashi, Tsukuba, Ibaraki 305-8565 Japan

**Keywords:** Influenza virus, Ultraviolet rays, Long-range RT-qPCR, Infectivity, Strand break, Water treatment

## Abstract

**Background:**

The polymerase chain reaction (PCR) is commonly used to detect viral pathogens because of its high sensitivity and specificity. However, conventional PCR methods cannot determine virus infectivity. Virus infectivity is conventionally examined with methods such as the plaque assay, even though such assays require several days. Long-range reverse-transcription quantitative PCR (RT-qPCR) has previously been suggested for the rapid assessment of RNA virus infectivity where the loss of infectivity is attributable to genomic fragmentation.

**Methods:**

IAV was irradiated with 253.7 nm ultraviolet (UV) rays to induce genomic strand breaks that were confirmed by a full-length RT-PCR assay. The IAV was then subjected to plaque assay, conventional RT-qPCR and long-range RT-qPCR to examine the relationship between infectious titer and copy number. A simple linear regression analysis was performed to examine the correlation between the results of these assays.

**Results:**

A long-range RT-qPCR assay was developed and validated for influenza A virus (IAV). Although only a few minutes of UV irradiation was required to completely inactivate IAV, genomic RNA remained detectable by the conventional RT-qPCR and the full-length RT-PCR for NS of viral genome following inactivation. A long-range RT-qPCR assay was then designed using RT-priming at the 3′ termini of each genomic segment and subsequent qPCR of the 5′ regions. UV-mediated IAV inactivation was successfully analyzed by the long-range RT-qPCR assay especially when targeting PA of the viral genome. This was also supported by the regression analysis that the long-range RT-qPCR is highly correlated with plaque assay (Adjusted *R*^*2*^ = 0.931, *P* = 0.000066).

**Conclusions:**

This study suggests that IAV infectivity can be predicted without the infectivity assays. The rapid detection of pathogenic IAV has, therefore, been achieved with this sensing technology.

## Background

Various techniques for virus detection have been developed [1–12]. Polymerase chain reaction (PCR) is one of the most commonly used methods to detect viruses in specimens because of its rapidity and high sensitivity [4, 7, 8]. With the recent growing demand for the rapid diagnosis of viral infection, novel techniques and devices based on PCR have been developed [13, 14]. These newer methods mostly shorten the assay time and reduce the proportion of false negatives. Despite these advances in PCR technology, PCR still cannot clearly discriminate between infectious and non-infectious organisms [15]. Virus infectivity is conventionally measured with infection studies incorporating methods such as the plaque assay or focus assay using cell cultures or animals [1, 4, 16]. Although these studies take several days to complete, they remain in common use for examining virus infectivity because of their reliability [17]. However, both reliability and rapidity are required for on-site inspections at the places such as hospitals, water treatment plants, and food plants. Alternative strategies for infectivity evaluation have previously been suggested in studies of both DNA (adenovirus, parvovirus, hepatitis B virus) and RNA (norovirus, MS2 bacteriophage) viruses [18–22]. Notably, long-range quantitative PCR (qPCR) was commonly employed in each of these studies. Long-range qPCR methods basically aim to exclude degraded genomes from the samples being tested as these nucleic acids are derived from viral particles that are no longer infective. In the case of DNA viruses, genomic DNA is pre-amplified by long-range PCR to prepare the 4–6 kbp PCR template required for the subsequent nested qPCR [19, 21]. Failure of viral DNA detection or decreased signal with the nested qPCR reflects the degree of genomic damage. As for RNA viruses, long-range reverse-transcription (RT) is first carried out to prepare complementary DNA (cDNA) prior to qPCR [18, 20]. Subsequent qPCR needs to take place at a locus that is separate from the RT-priming site to reduce the influence of fragmented RNA. This method was named long-range RT-qPCR. These genomic damages represent the inability of viral replication. Ultraviolet (UV) rays are the powerful sterilizing sources that introduce genomic damages in pathogens [23]. A maximum sterilizing effect of UV rays on pathogens are shown at 253.7 nm [23, 24].

Influenza A virus (IAV) is classified as part of the *Orthomyxoviridae* family and its genome comprises eight segments of single-stranded, negative-sense RNA [25]. IAV is one of the pathogens of most concern in healthcare as it causes a cold-like illness with symptoms that can be particularly severe in the elderly and infants [26]. IAV occasionally recombines its genes among the different subtypes, resulting in the generation of new subtypes [27]. These emerging subtypes can potentially cause pandemics because no population has ever experienced some of the antigens on these viruses [27]. In the pandemic situations, quick assessment of IAV infectivity, such as long-range RT-qPCR, is quite important to build a strategy for preventing spread of infection. The longest IAV genomic segments are approximately 2.3 kb in length and comprise segments 1 (PB2), 2 (PB1), and 3 (PA), which encode subunits of an RNA-dependent RNA polymerase [25]. The RNA polymerase plays an essential role in virus genome replication, which requires the interaction of each subunit. Therefore, strand breaks in these genomic segments should be correlated with IAV inactivation. A long-range RT-qPCR with a PCR site 2.3 kb upstream of the RT-priming site was used to predict human norovirus GI and GII infectivity in a previous study [20]. The results indicated that long-range RT-qPCR would potentially be applicable to the assessment of IAV infectivity by targeting PB2, PB1 and PA.

In this study, we focused on the development of a rapid testing method for examining IAV infectivity without the use of cell culture or animals. Here, therefore, we investigated whether long-range RT-qPCR is applicable to determination of IAV infectivity. We also appraised the proper length of the target for long-range RT-qPCR by comparing the results for each segment.

## Methods

### Cell culture and virus preparation

Madin-Darby Canine Kidney (MDCK) cells (accession no. CCL-34, ATCC, Manassas, VA) were maintained at 37 °C and 5% CO_2_ in the culture media [Dulbecco’s Modified Eagle’s Medium (Fujifilm Wako Pure Chemical Corporation, Osaka, Japan) supplemented with 10% fetal bovine serum (Fujifilm Wako Pure Chemical Corporation), 100 U/mL penicillin, and 100 μg/mL streptomycin (Fujifilm Wako Pure Chemical Corporation)]. IAV (Panama/2007/99/H3N2) was propagated in the MDCK cells. Culture supernatant aliquots were stored as IAV stocks at − 80 °C prior to use. The titer of the IAV stocks was determined by plaque assay as described below.

### UV irradiation of IAV

The IAV stocks were irradiated with a UV lamp with a wavelength and fluence of 253.7 nm and 12.5 μW/cm^2^, respectively, to introduce strand breaks in the virus genome. UV-irradiated IAV was subjected to plaque assay and RNA isolation. The effect of UV irradiation on IAV infectivity was examined by plaque assay as described above. Isolated RNA was used for RT-PCR analyses.

### Plaque assay

IAV infectious titer was analyzed by plaque assay according to a previous report [4] with slight modifications. Briefly, MDCK cells were split into 6-well culture plates at the concentration of 5 × 10^5^ cells/well. An aliquot of IAV stocks was thawed and 10-fold serially diluted in the culture media. MDCK cells were inoculated with 400 μL of each IAV dilution and incubated at 37 °C for 1 h with rocking every 15 min. The inoculum was removed and the cells were washed once with phosphate-buffered saline (−) to eliminate the unbound virus particles. The cells were overlaid with overlay media [KM220 (Kohjin-bio, Saitama, Japan) containing 1.25 μg/mL acetylated trypsin (Sigma-Aldrich Japan, Tokyo, Japan) and 0.8% (w/v) SeaPlaque agarose (Lonza Japan K.K., Tokyo, Japan)] and additionally incubated at 37 °C for 72 h. Cells were fixed and stained in 20% ethanol containing 1% crystal violet at room temperature for 15 min to visualize the plaques. The plaque number was counted and multiplied by the dilution ratio to calculate the infectious titer. Infectious titers were defined as the number of plaque-forming units (pfu)/mL and data represent the mean ± standard error of four independent experiments.

### Conventional RT-qPCR

A conventional RT-qPCR assay was conducted to measure the IAV copy number. Viral RNA was isolated from 140 μl virus suspension using the QIAamp Viral RNA Mini Kit (Qiagen K.K., Tokyo, Japan) according to the manufacturer’s instructions. Each RNA was eluted in 60 μl of an elution buffer. The isolated RNA was immediately subjected to RT-qPCR with the TaqMan Fast Virus 1-Step Master Mix (Thermo Fisher Scientific K.K., Tokyo, Japan), a TaqMan probe (5′-FAM-ATYTCGGCTTTGAGGGGGCCTG-MGB-3′), and a primer pair (Forward: 5′-CCMAGGTCGAAACGTAYGTTCTCTCTATC-3′, Reverse: 5′-TGACAGRATYGGTCTTGTCTTTAGCCAYTCCA-3′). The probe and primer sequences were derived from the IAV diagnosis manual released by the World Health Organization (WHO) [28]. Thermal cycling was carried out on the LightCycler 96 (Roche Diagnostics K.K., Tokyo, Japan) for reverse transcription at 50 °C for 5 min, denaturation of the RT polymerase at 95 °C for 20 s, and 40 cycles of PCR at 95 °C for 3 s and 60 °C for 30 s. Copy number quantification was carried out with an IAV standard curve. In brief, for the standard sample preparation, each PCR fragment was purified from agarose gel electrophoresis using QIAquick Gel Extraction kit (Qiagen K.K.), measured the concentration by a spectrophotometer Nanodrop (Thermo Fisher Scientific K.K.), then determined the copy number using DNA Copy Number and Dilution Calculator (Thermo Fisher Scientific K.K.). These standard samples were 10-fold serially diluted and subjected to the real-time RT-qPCR analyses at the same time with the IAV samples to make standard curves. Copy numbers of each time point was divided by that of 0 min (No UV) to calculate the copy number ratio. Assay was performed in a triplicate experiment.

### Long-range RT-qPCR

PB2, PB1, and PA were analyzed by long-range RT-qPCR to evaluate the effect of UV irradiation. RNA was identically isolated from virus suspension and reverse-transcribed using SuperScript III (Thermo Fisher Scientific K.K.) with an RT primer (5′-AGCGAAAGCAGG-3′) that specifically primed at the 3′ termini of PB2, PB1, and PA of IAV (Panama/2007/99/H3N2) [29]. qPCR was performed using Power SYBR Green PCR Master Mix (Thermo Fisher Scientific K.K.) according to the manufacturer’s instructions. Thermal cycling was carried out on the LightCycler 96 for activation of the DNA polymerase at 95 °C for 10 min, and 40 cycles of PCR at 95 °C for 15 s and 60 °C for 1 min. qPCR primers for each segment were designed with Primer3Plus (http://www.bioinformatics.nl/cgi-bin/primer3plus/primer3plus.cgi) to amplify the region more than 2 kb away from the RT-priming sites. Segments 4 to 8 (HA, NP, NA, M, and NS) were similarly examined by long-range RT-qPCR, with an RT primer (5′-AGCAAAAGCAGG-3′) and the qPCR primers for each segment designed by Primer3Plus to amplify the region within 250 bp of the 5′ termini. All of the qPCR primer sequences are available upon request. A denaturing protocol was carried out in the RT-qPCR to check the denaturing temperature of PCR products, that is commonly employed to verify the primer specificity. Quantification of the copy numbers was performed with standard curves as shown in the subsection of conventional RT-qPCR. Values below that of the most diluted standard were defined as “not detected” and included in the datasets as 0. The cut-off copy numbers were considered as below 2.35, 44.2, 1.95, 1.77, 4.45, 1.08, and 7.80 copies/μl for PB2, PA, HA, NP, NA, M, and NS, respectively. Copy number ratio was calculated as shown in the subsection of conventional RT-qPCR. Assays were conducted in triplicate experiments and repeated for three times.

### Full-length RT-PCR

The degrees of RNA degradation of PA and NS were examined by full-length RT-PCR. Same cDNAs prepared in the long-range RT-qPCR were subjected to RT-PCR using PrimeStar Max DNA polymerase (TaKaRa, Shiga, Japan) according to the manufacturer’s instruction. Thermal cycling was carried out on the MiniAmpPlus Thermal Cycler (Thermo Fisher Scientific K.K.) for activation of the DNA polymerase at 98 °C for 1 min, 40 cycles of PCR at 98 °C for 10 s, 55 °C for 5 s and 72 °C for 2 min, and for deactivation of the DNA polymerase at 72 °C for 7 min. Primer sets were designed to amplify most of the segments of PA (2105 bp of 2338 bp, Forward: 5′-GCTTCAACCCGATGATTGTC-3′, Reverse: 5′-GGAGTTGAACCAAGACGCAT-3′) and NS (558 bp of 887 bp, Forward: 5′-GAACTGAGTGATGCCCCATT-3′, Reverse: 5′-TCCCCCATTCTCATTACTGC-3′) which include the regions of the long-range RT-qPCR.

### Statistics

The significances of all the datasets in plaque assay, the conventional RT-qPCR, and the long-range RT-qPCRs were verified by the Kruskal-Wallis test. Student’s t-test was conducted to assess the significance between specific two different datasets of averaged technical replicates in the long-range RT-qPCRs and plaque assay. A simple linear regression analysis was conducted using IBM SPSS Statistics (IBM Japan Ltd., Tokyo, Japan) to examine the significant correlation between the plaque assay and each long-range RT-qPCR as well as the conventional RT-qPCR. All the significances were considered to have *P* < 0.05.

## Results

### Effect of UV irradiation on IAV infectivity

**Fig. 1:**
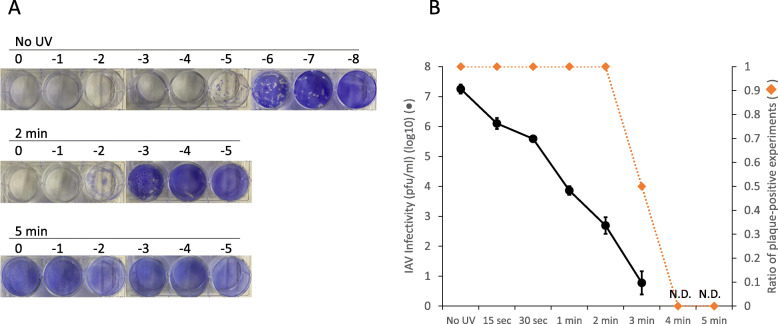
Examination of the effect of UV irradiation on IAV infectivity by plaque assay. IAV suspensions were irradiated with a UV lamp for increasing periods of time between 15 s to 5 min. IAV infectivity was measured by plaque assay using MDCK cells. **a** The representative pictures of plaques of No UV, 2 min, and 5 min are shown. Dilution factors of IAV are displayed on the top of each well. **b** The titers are plotted on the graph and shown as pfu/mL (●). Assays were repeated four times and values represent the mean ± standard error. No plaque formation was observed after 4 and 5 min of irradiation and these were defined as not detected (ND). The ratio of experiments in which plaque formation was observed is also plotted on the graph (◆)

IAV stocks were irradiated with a UV lamp for increasing periods of time from 15 s to 5 min and the plaque assay was then conducted to determine the required length of time for UV irradiation to completely inactivate the IAV stocks (Fig. 1a and b). The infectivity decreased with increasing length of UV exposure. The original titer of 2.2 × 10^7^ pfu/mL (No UV) dropped to 1.9 × 10 pfu/mL by 3 min of irradiation (*P* < 0.05). IAV replication completely ceased after 4 min or more of UV irradiation (*P* < 0.05, 4 min vs. No UV). Even after 3 min of irradiation, plaques were not observed in two of the four replicate experiments. The not observed values were considered as 0 pfu/ml and included in the calculation for titers. These results indicated that 4 min of UV irradiation was sufficient to inactivate the IAV stocks.

### Influence of UV irradiation on strand breaks in the IAV genome

**Fig. 2:**
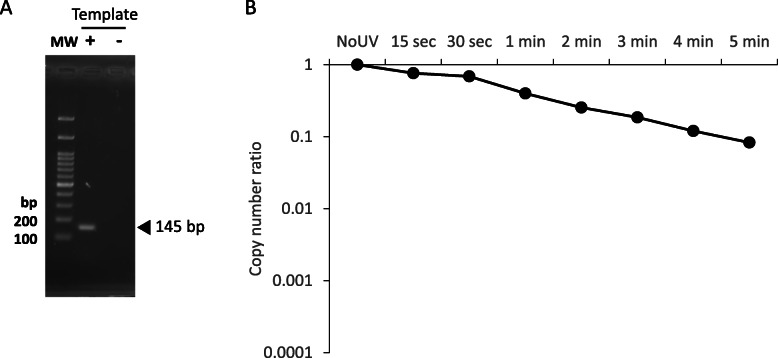
Induction of strand breaks in the IAV genome by UV exposure. IAV suspensions were irradiated with a UV lamp for increasing periods of time between 15 s to 5 min. **a** Representative PCR product (145 bp) was visualized by agarose gel electrophoresis to show the primer specificity. MW; Molecular weight marker (**b**) IAV copy number was then measured by conventional RT-qPCR. Assays were carried out in triplicate and values represent the mean ± standard error

IAV copy number in the UV-irradiated virus suspensions was quantified by RT-qPCR to evaluate the degree of genomic RNA degradation (Fig. 2). Here, we used the primer and probe set suggested by the WHO for IAV detection, which targets M of IAV genomic segment. The PCR product size and primer specificity were verified in the agarose-gel electrophoresis (Fig. 2a). IAV copy number and the infectious titer decreased with longer UV exposure (Fig. 2b). Approximately 90% of the genome was reduced after 4–5 min of UV exposure (*P* < 0.001), at which point IAV lost infectivity. This result suggested that the loss of infectivity caused by UV irradiation was associated with genomic strand breaks as reported previously [30]. However, genomic RNA was still detectable by RT-qPCR. Even after overnight UV irradiation, the IAV genome was faint but still detected with a small copy number (data not shown). While the conventional RT-qPCR employed here is useful to detect and quantify IAV in specimens as outlined in the WHO guidelines, other approaches such as long-range RT-qPCR need to be developed to assess IAV infectivity.

### Eligibility of long-range RT-qPCR to assess IAV infectivity

**Fig. 3:**
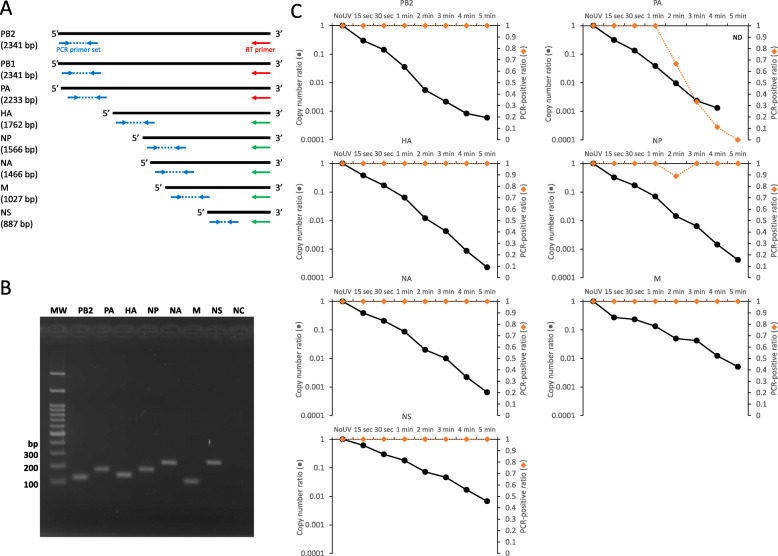
Long-range RT-qPCR to determine IAV infectivity. **a** Schematic representation of IAV genomic segments, RT-priming sites, and qPCR sites. Common RT primers for segments 1–3 and segments 4–8 are indicated with red and green arrows, respectively. The PCR primers were specific for each segment. **b** Representative PCR products were visualized by agarose gel electrophoresis to show specificities of the primer pairs. PCR product sizes are 117, 159, 137, 171, 226, 114, and 231 bp for PB2, PA, HA, NP, NA, M, and NS, respectively. MW; Molecular weight marker, NC; No primer control (**c**) The IAV suspension was irradiated with a UV lamp for increasing periods of time between 15 s to 5 min. IAV copy number was measured by the long-range RT-qPCR assay developed in the present study. Assays were repeated in triplicate and values represent the mean ± standard error (●). The PCR-positive ratio is also plotted on the graph (◆)

The lengths of IAV (Panama/2007/99/H3N2) PB2 (DQ487334), PB1 (DQ487333), PA (DQ487335), HA (DQ487340), NP (DQ487339), NA (DQ487337), M (DQ487338), and NS (DQ487336) are 2341, 2341, 2233, 1762, 1566, 1466, 1027, and 887 bp, respectively, according to the NCBI GenBank (Fig. 3a). We hypothesized that long-range RT-qPCR would be applicable to at least PB2, PB1, and PA because the distances between the RT-priming sites and the qPCR sites were equivalent to that for human norovirus, which was previously successfully subjected to long-range RT-qPCR. The RT reactions were primed at the 3′ termini using a common RT primer (5′-AGCGAAAGCAGG-3′), followed by qPCR with specific primer pairs for each segment (Fig. 3a). The PCR product size and primer specificity were confirmed by the agarose-gel electrophoresis (Fig. 3b). qPCR was successful for PB2 and PA but not for PB1 with any of the primer pairs we designed (Fig. 3c). We, therefore, did not include PB1 in any further analyses. The copy number was reduced with increasing length of UV irradiation and approximately 99.9% of the copies were fragmented by 4 min for both PB2 and PA (*P* < 0.001) (Fig. 3c). In particular, PA was detectable only in three, one, and zero replicates out of nine at the time points of 3, 4, and 5 min, respectively, while PB2 was still detectable in every replicate at each time point. Next, we also performed long-range RT-qPCR for the other segments to evaluate their importance in the assay. A common RT primer (5′-AGCAAAAGCAGG-3′) and specific primer pairs were used for each segment (Fig. 3a). The PCR products were visualized by electrophoresis as well as PB2 and PA (Fig. 3b). Approximately 99.9% or more of HA, NP, and NA were degraded by 4 min similar to PB2 and PA (*P* < 0.001) (Fig. 3c). In contrast, the reduction ratio of M and NS was below 99% at 4 min (*P* < 0.001). A simple linear regression analysis was conducted to examine the correlation between the results of plaque assay and each long-range RT-qPCR as well as the conventional RT-qPCR (Table 1). All the results obtained in the RT-qPCRs were significantly correlated with the plaque assay (*P* < 0.05). In particular, the long-range RT-qPCR for PB2 and PA showed the highest significant values (PB2: adjusted *R*^*2*^ = 0.938, *P* = 0.000049, PA: adjusted *R*^*2*^ = 0.931, *P* = 0.000066) among them. This indicates that the long-range RT-qPCR for PB2 and PA are mostly correlated with the plaque assay and the importance of the lengths of genome.

**Table 1:** Simple linear regression analysis to examine the correlation between plaque assay and the RT-qPCRs

	Adjusted R2	F	*P-value*
PB2	0.938	106	0.000049
PA	0.931	95.5	0.000066
HA	0.888	56.7	0.000283
NA	0.92	81.4	0.000103
NP	0.875	49.9	0.000403
M	0.928	90.5	0.000077
NS	0.861	17.2	0.006
Conventional RT-qPCR	0.699	6.2	0.047

Plaque assay and RT-qPCRs were considered as dependent and independent variables, respectively. PB2, PA, HA, NA, NP, M, and NS indicates the long-range RT-qPCR for PB2, PA, HA, NA, NP, M, and NS, respectively. Adjusted R2: Adjusted correlation coefficient

**Fig. 4:**
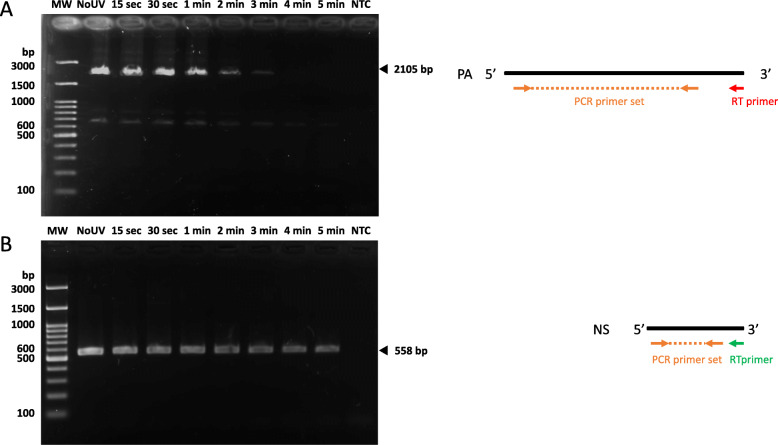
Full-length RT-PCR analysis to compare the genomic degradation efficiency by UV irradiation. Same cDNA sets used in the long-range RT-qPCR were analyzed with the full-length RT-PCR for (**a**) PA and (**b**) NS. The PCR products were visualized by agarose-gel electrophoresis in the left panel. The schematic representation of the full-length RT-PCR was shown in the right panels. PCR and RT primers are indicated as in Fig. 3a. The PCR product sizes are 2105 bp and 558 bp for PA and NS, respectively. MW; Molecular weight marker, NTC; No template control

For further confirmation that the RNA degradation by UV is size dependent, we tried to visualize the initial RNA directly; however, the RNA amount was too small to visualize in agarose-gel electrophoresis. Therefore, we conducted full-length RT-PCR for PA and NS to compare the efficiency of RNA degradation by UV (Fig. 4). This assay shows the existence and amount of full-length cDNA which reflect the degradation degree of initial viral RNA. Same cDNA sets used in the long-range RT-qPCR were subjected to the full-length RT-PCR using PCR primer pairs to amplify 2105 bp and 558 bp for PA and NS, respectively (Fig. 4a and b). Both of PCR products were successfully amplified in No UV sample and reduced as increase of UV exposure time. PA was clearly amplified until 3 min, faintly appeared at 4 min, and completely disappeared at 5 min, while NS was robustly detected in all the samples. These results coincide with the long-range RT-qPCR and indicate that the RNA degradation efficiency is size dependent.

Finally, we compared the sensitivities of the long-range RT-qPCR and the conventional RT-qPCR with the plaque assay (Table 2). The comparison was carried out as follows: the original (No UV) copy number measured in the long-range RT-qPCR of each segment and the conventional RT-qPCR was divided by the original titer of the plaque assay, then the percentages are shown as the normalized sensitivity of the long-range RT-qPCR and the conventional RT-qPCR. Normalized sensitivities against the plaque assay range from 24% (PA) to 43,636% (the conventional RT-qPCR). Interestingly, only the sensitivity of the long-range RT-qPCR for PA was lower than the plaque assay, while the other RT-qPCRs were more sensitive than that. This indicates that these RT-qPCRs except for the long-range RT-qPCR for PA potentially detects extra non-infectious IAVs that are not detectable in the plaque assay. In other words, only the long-range RT-qPCR for PA is highly specific for infectious IAVs.

**Table 2:** Comparison of sensitivities of the long-range RT-qPCR with the conventional RT-qPCR and the plaque assay

	RT-qPCR (copies/ml)	Normalized to plaque assay (%)
PB2	5.7 × 107	261
PA	5.4 × 106	24
HA	5.3 × 108	2395
NA	6.8 × 108	3109
NP	4.5 × 108	2041
M	3.6 × 108	1627
NS	1.5 × 108	700
Conventional RT-qPCR	9.6 × 109	43,636

Original copy numbers measured in the RT-qPCRs were divided by that in the conventional RT-qPCR or the titer in the plaque assay. Values are shown as percentages. PB2, PA, HA, NA, NP, M, and NS indicates the long-range RT-qPCR for PB2, PA, HA, NA, NP, M, and NS, respectively. Normalization was performed as follows: RT-qPCR (copies/ml) / Plaque assay (2.2 × 107 pfu/ml)

## Discussion

The rapid detection of pathogens is in high demand for the purpose of on-site inspection of specimens at hospitals and of treated water at water treatment plants [31, 32]. In healthcare settings, patients with flu symptoms are usually diagnosed with an IAV diagnostic test using immunochromatography with antibodies against IAV antigens [31]. This rapid antigen test is inexpensive and can be completed within 20–30 min. However, its sensitivity is much lower than that of other tests [3]. We previously showed that an immunochromatographic test commonly used at hospitals in Japan was 10^5^-fold less sensitive than qPCR [3]. This indicates that the immunochromatographic test would frequently show false negatives because the IAV copy number can be less than the detection limit of the assay [6]. Indeed, a clinical study showed that most patients are misdiagnosed as negative for influenza virus based on initial testing using immunochromatography [33]. Alternative methods such as RT-qPCR on Roche’s cobas Liat System have been developed to address this issue [14]. These newer rapid diagnostic tests target IAV genomic RNA instead of viral antigens. This provides not only quicker results turnaround but also sufficient accuracy and sensitivity for hospital-based diagnosis. In addition, this type of test can determine the IAV subtypes responsible by sensing subtype-specific sequences [34]. These are major advantages for public health in terms of preventing epidemics. Recent improvements in UV light-emitting diodes (LED) technology will likely accelerate the installation of UV sanitization where UV irradiation has not been used for this purpose [35]. LEDs are expected to reduce the running costs of UV-based sanitization because of their lower electricity consumption and longer lifespan [35]. A previous study emphasized the importance of UV LED in disinfection for pathogens not only at water treatment plants but throughout the entire water distribution system [36]. This is because a number of contamination cases are considered to have occurred as a result of deficiencies in the distribution system. Some of the highly pathogenic avian influenza viruses, such as the H5N1 and H9N2 subtypes included in IAV, are also considered to be partially transmitted to humans through the consumption of contaminated water [37, 38]. In response to this trend, methods that can be used to verify successful disinfection in real time are desirable [32]. The rapid molecular test based on long-range RT-qPCR described in the present study may represent such a solution for this issue.

Again, this study focused on the development of a rapid testing method for examining IAV infectivity without the use of cell culture or animals. We utilized UV irradiation to inactivate IAV by introducing strand breaks into the genomic RNA segments. Viral replication was completely abolished after a few minutes of UV irradiation with degradation of the viral RNA as expected (Fig. 1). However, viral RNA was still detectable even though no infectious IAV was contained in the virus suspensions (Fig. 2). This result agrees with that of a previous study where short wavelength (280 nm) UVC-LED was effective for IAV inactivation based on a plaque assay but where viral genomic RNA continued to yield a positive result by RT-qPCR [30]. In this case, the qPCR site was designed 150 bp upstream of the RT-priming site. In our study, we did not use any specific RT-primers for the conventional RT-qPCR because the commercial RT-qPCR kit we used contained random primers. Random primers are generally considered to bind to most templates. For this reason, a number of cDNAs containing the PCR target sequence were likely also synthesized from adjacent sites. The distance between the qPCR site and the RT-priming site is an important factor in the detection of RNA virus infectivity [18, 20]. If this distance is too long, more individual viruses will fail to be transcribed into cDNA with no amplification as a result. Here, we showed that long-range RT-qPCR for each genomic segment produced a similar trend in the infectivity results. PB2, PA, HA, NP, and NA with lengths ranging from 1.5 to 2.3 kb showed between 99 and 99.9% degradation after 4–5 min of irradiation, at which point the virus particles became non-infective. In contrast, M and NS, which are 1 kb or less in length, reached less than 99% reduction after 4–5 min. Based on these observations, it may be good practice to design the qPCR sites more than 1.5 kb away from the RT-priming site to more clearly observe genomic fragmentation. In particular, PA provides the most accurate indication of the degree of infectivity following UV irradiation (Fig. 3 and Table 1). Fluctuation in the PCR results (66.7% negative) at the time point of 3 min (Fig. 3c for PA) was similar to that observed for the infectivity study in which 50% of the virus suspensions lost infectivity (Fig. 1b). The positive result obtained for one of the nine replicates at 4 min was not statistically significant when compared with the data at 5 min (student’s t-test, *P* = 0.346). This indicates that non-infective virus particles that have genomic fragmentation in segments other than PA would occasionally be detected in the assay with a low frequency. This is also supported by the regression analysis and sensitivity analysis in this study (Tables 1 and 2). Unexpectedly, PB2, which is almost the same length as PA, still exhibited a high proportion of positive results at 4 and 5 min among the replicates. The other segments showed similar results. This might have been the result of differences in RT efficiency between segments rather than an effect of the different segment lengths, although we have not yet fully elucidated this.

The discrepancy between virus copy number and infectivity titer has been often observed in many studies [3, 39]. This is apparently attributed to the presence of defective virions and virus aggregation [39, 40]. Defective virions are unable to infect for variable reasons including genomic deletions and mutations, lacking receptor-binding ability, and disruption of particles, however, these virions can be positive in PCR-based analysis as long as they retain the target region of PCR [39]. Viruses prepared in cell culture tend to aggregate each other to form big clumps of multiple virions that seem to correspond to single plaque [41]. This reduces the apparent infectivity of the virus preparation even with the presence of extra infectious virions. These are the reason why IAV genome was detected both in the long-range RT-qPCR and the conventional RT-qPCR even after the virus lost infectivity by UV exposure. In other words, we might have detected the genomes of defective virions or extra infectious virions of the clumps that were not involved in forming plaques. Indeed, it took us for 24 h of UV exposure to diminish most of the detectable genomes in the conventional RT-qPCR (data not shown). This might be occurred because the conventional RT-qPCR was too sensitive. Our long-range RT-qPCR for every segment was much less sensitive than the conventional RT-qPCR (Table 2). Particularly, sensitivity of the assay for PA was even lower than plaque assay, which might have reduced the chance to detect non-infectious viruses and increased its specificity for infectious viruses. The detailed studies for this aspect will provide important insight into the successful long-range RT-qPCR establishment.

Although we demonstrated that the long-range RT-qPCR targeting PA can be a useful tool to examine the infectivity without cell culture, some of issues remain to be solved. One of the biggest concerns is how the assay is applicable for the other IAV subtypes. This is determined by how the primer binding sites are conserved among the subtypes. The RT-primer suits most of the cases because its binding site is highly conserved across the IAV subtypes [29]. As for the qPCR primers, our in silico analysis suggested that the primer set for PA of the long-range RT-qPCR potentially binds to the other subtypes (H1N1, H2N2, H5N1, H7N9, H9N2) because of the homology of the primer binding sequences (1–5 and 0–1 mismatches out of 20-mer in Forward or Reverse primers, respectively) (data not shown). However, further experiments are required to determine the appropriate primer set for all the IAV subtypes.

## Conclusions

In summary, our study indicated that IAV infectivity can be predicted by long-range RT-qPCR targeting PA, in particular, to determine the effect of UV irradiation. Long-range RT-qPCR of the other segments can also provide helpful information to support the results from PA. Although additional studies are required, our findings may assist in the future development of IAV prevention strategies.

## Data Availability

All the dataset generated or analyzed during the current study are available from the corresponding author upon request.

## Abbreviations

PCR: Polymerase chain reaction
RT-qPCR: Reverse-transcription quantitative polymerase chain reaction
IAV: Influenza A virus
UV: Ultraviolet
MDCK: Madin-Darby Canine Kidney
